# Trends and Disparities in the Use of Next-Generation Sequencing in Patients With Cancer in the United States

**DOI:** 10.1001/jamanetworkopen.2026.5585

**Published:** 2026-04-07

**Authors:** Chadi Hage Chehade, Zeynep Irem Ozay, Yeonjung Jo, Georges Gebrael, Nicolas Sayegh, Micah Ostrowski, Ayana Srivastava, Diya Garg, Tanner Hardy, Ethan G. Murdock, Arshit Narang, Vinay Mathew Thomas, Haoran Li, Benjamin L. Maughan, Soumyajit Roy, Neeraj Agarwal, Umang Swami

**Affiliations:** 1 Division of Medical Oncology, Department of Internal Medicine, Huntsman Cancer Institute, University of Utah, Salt Lake City; 2 Cancer Biostatistics, Huntsman Cancer Institute, University of Utah, Salt Lake City; 3 Department of Internal Medicine, University of Texas Southwestern Medical Center, Dallas; 4 Division of Medical Oncology, Department of Internal Medicine, University of Kansas Cancer Center, Westwood; 5 Department of Radiation Oncology, University Hospitals Seidman Cancer Center, Case Western Reserve University, Cleveland, Ohio

## Abstract

**Importance:**

Life-prolonging targeted therapies are available based on alterations detected on next-generation sequencing (NGS); however, clinical data on NGS adoption are limited.

**Objective:**

To assess trends and disparities in NGS among patients with common advanced or metastatic cancers.

**Design, Setting, and Participants:**

Retrospective cohort study of 280 US-based cancer clinics (approximately 800 sites of care) using electronic health record–derived deidentified information from Flatiron Health Research Database. Participants were patients diagnosed with metastatic breast (mBC), metastatic prostate (mPC), advanced non–small cell lung (aNSCLC), metastatic colorectal (mCRC), and metastatic pancreatic (mPanC) cancers between January 1, 2018, and December 30, 2022. Data were analyzed from April 2024 to December 2025.

**Main Outcomes and Measures:**

Time to NGS from diagnosis, considering death as competing risk. Multivariable cause-specific Weibull accelerated failure time models evaluated association of socioeconomic status (SES), race and ethnicity, insurance type, practice setting, and sex with time to NGS.

**Results:**

Overall, 63 294 patients with advanced or metastatic cancer were eligible and included: 12 085 with mBC (19.1%), 4341 with mPC (6.9%), 27 050 with aNSCLC (42.7%), 13 648 with mCRC (21.6%), and 6170 with mPanC (9.7%). The median (IQR) age was 68 (60-76) years and 33 975 patients (53.7%) were female; 1703 (2.7%) were Asian, 6551 (10.0%) were Black, 3772 (6.0%) were Hispanic, 38 318 (61.0%) were White, and 12 950 (20.3%) were other races and ethnicities. One-year cumulative incidence of NGS increased in patients diagnosed in 2022 compared with 2018 across all cancer types. Significantly longer time to NGS (time ratio [TR]) was observed in those with mBC with low SES (1, lowest: TR, 1.3; 95% CI, 1.1-1.6; 2: TR, 1.3; 95% CI, 1.1-1.5), those who were Hispanic (TR, 1.4; 95% CI, 1.2-1.7), or those on Medicare (TR, 1.5; 95% CI, 1.3-1.8). Longer TRs were also observed in those with mPC and Hispanic ethnicity (TR, 1.6; 95% CI, 1.3-2.1), those on Medicaid (TR, 2.0; 95% CI, 1.2-3.5), those with aNSCLC with low SES (1, lowest: TR, 1.6; 95% CI, 1.5-1.8; 2: TR, 1.4; 95% CI, 1.3-1.6; 3: TR, 1.2; 95% CI, 1.1-1.3), Black patients (TR, 1.4; 95% CI, 1.2-1.5), and those on Medicare (TR, 1.4; 95% CI, 1.3-1.5). For mCRC, TRs were elevated for those with low SES (1, lowest: TR, 1.3; 95% CI, 1.1-1.5; 2: TR, 1.1; 95% CI, 1.0-1.3), Black patients (TR, 1.4; 95% CI, 1.2-1.6), Hispanic patients (TR, 1.4; 95% CI, 1.2-1.6), and those on Medicare (TR, 1.4; 95% CI, 1.3-1.6). Elevated TRs were also observed for mPanC in Black patients (TR, 1.5; 95% CI, 1.2-1.8) and those on Medicare (TR, 1.3; 95% CI, 1.1-1.5).

**Conclusions and Relevance:**

In this cohort study, many patients with advanced or metastatic cancers did not undergo tumor genomic testing. Low SES, Black race or Hispanic ethnicity, and Medicaid or Medicare coverage were associated with significantly longer time to NGS, highlighting the need for awareness and health care policies aimed at bridging these gaps.

## Key Points

**Question:**

What are the trends and disparities in the use of next-generation sequencing testing among patients with advanced cancers in the US?

**Findings:**

In this cohort study of 63 294 patients with advanced or metastatic breast, prostate, colorectal, pancreatic, and lung cancers, many patients did not undergo tumor genomic testing. Patients with low socioeconomic status, Black or Hispanic patients, and those covered by Medicaid or Medicare experienced significantly longer time to testing in some of these cancers compared with their respective reference groups.

**Meaning:**

These findings suggest disparities persist in genomic testing and underscore the need for interventions to ensure equitable access to precision oncology.

## Teaser

This cohort study evaluates trends and disparities in the use of next-generation sequencing among patients with common advanced and/or metastatic cancers.

## Introduction

Next-generation sequencing (NGS) emerged as a transformative technology, enabling comprehensive genomic profiling that drives precision medicine.^1^ By uncovering actionable alterations, NGS enables informed selection of targeted therapies shown to improve survival outcomes in patients with advanced cancer.^2,3^ Additionally, NGS plays a crucial role in detecting biomarkers that guide prognostication in the clinic and determining eligibility for clinical trials.^4,5^

In 2024, breast, prostate, lung, colorectal, and pancreatic cancers were the most frequently diagnosed advanced neoplasms in the US.^6^ Current guidelines recommend NGS to guide treatment selection in the clinic for these cancers, marking a shift in the recommendations compared with 2018. At that time, NGS guided treatment for most patients with refractory cancers, while it was still used in frontline treatment decisions for certain cancers, such as non–small cell lung cancer.^7^ In metastatic prostate cancer (mPC), poly(ADP)-ribose polymerase (PARP) inhibitors have been approved as a life-prolonging therapeutic option in patients with homologous recombination repair (HRR) alterations.^8,9^ In patients with metastatic breast cancer (mBC), NGS can uncover alterations in *PIK3CA*, *ESR1*, and *BRCA1/2*, for which approved therapies exist.^10,11,12^ In metastatic colorectal carcinoma (mCRC), NGS enables identification of alterations in *KRAS*, *NRAS*, and *BRAF* that could guide treatment selection with cetuximab (anti-EGFR monoclonal antibody).^13^ Patients with platinum-sensitive metastatic pancreatic cancer (mPanC) harboring germline *BRCA1/2* alterations can receive maintenance treatment with olaparib (PARP inhibitor).^14^ Advanced non–small cell lung cancer (aNSCLC) has also seen a substantial increase in the number of targetable alterations, including *EGFR*, *KRAS*, *ALK*, *ROS1*, *BRAF*, *NTRK1/2/3*, *MET*, and *RET*.^15^ In addition to these cancer-specific drug approvals, there are currently multiple tumor-agnostic therapies based on genomic profiling, including those targeting *NTRK* fusions, *RET* fusions, *BRAF* alterations, microsatellite instability-high/mismatch repair deficiency, and high tumor mutational burden (≥10 alterations per megabase).^16^

Despite these advantages, the implementation of NGS in clinical practice exhibits significant variability across cancer types and demographic groups.^17^ We and others have previously shown that non-Hispanic Black or Hispanic patients, those with low socioeconomic status (SES), or those covered by Medicare or Medicaid were significantly less likely to undergo tumor genomic profiling across multiple malignant neoplasms.^18,19,20^ In this study, we sought to analyze trends in NGS tumor testing among patients with the most prevalent solid tumors in the US and the association between social determinants of health and access to testing.

## Methods

### Patient Selection

This cohort study received institutional review board approval at the University of Utah. Informed consent was waived because the data were deidentified. The study complied with US patient confidentiality regulations, including the Health Insurance Portability and Accountability Act of 1996, and followed the Strengthening the Reporting of Observational Studies in Epidemiology (STROBE) reporting guideline for cohort studies.

Patient-level data were extracted retrospectively from the US-based, electronic health record (EHR)–derived deidentified Flatiron Health Research Database. This longitudinal database includes structured and unstructured data collected through technology-enabled abstraction and supplemented with third-party mortality information.^21^ The deidentified data were sourced from approximately 280 cancer clinics, encompassing approximately 800 sites of care.

The analytic cohort included patients diagnosed with mBC, mPC, aNSCLC, mCRC, or mPanC between January 1, 2018, and December 30, 2022. Patients with 2 or more advanced malignant neoplasms or without first-line treatment information were excluded. The data cutoff date was January 31, 2023.

### Patient Exposures

Next-generation sequencing was performed on tumor tissue or circulating tumor DNA (ctDNA). We defined the NGS date as the date the testing result was delivered, or if unavailable, the date the specimen was received.

Social determinants of health were evaluated by race and ethnicity, which included Asian non-Hispanic, Black non-Hispanic, Hispanic, White non-Hispanic, and other (Alaska Native, American Indian, Native Hawaiian, Other Pacific Islander, or multiracial), SES (ranging from 1, lowest, to 5, highest), insurance plan (commercial health plan, Medicare or other government programs, Medicaid, or others), practice type (academic vs community), and sex (male vs female).

Race and ethnicity data were generally collected from patients during intake interviews and forms, with variability across practices. The area-level SES index was calculated using Census block group data from the 2015 to 2019 American Community Survey, following the Yost Index methodology.^22^ Population-standardized SES quintiles, based on the most recently documented patient residential address, were applied, ranging from 1 to 5, with 1 representing the lowest SES and 5 the highest.^23^

### Statistical Analysis

Baseline characteristics were summarized using frequency and percentages for categorical variables, and median (IQR) for continuous variables. We assessed the disparities in access to NGS by race and ethnicity (reference: White non-Hispanic), SES (reference: 5, highest quintile), insurance (reference: commercial health plan), practice type (reference: community practice), and sex (if applicable; reference: female). The primary end point was NGS after advanced or metastatic disease diagnosis, and death was considered a competing risk. Patients were censored at the time of data cutoff or loss to follow-up, defined as the last recorded date of structured activity. We estimated the cumulative incidence functions (CIFs) for time to NGS by cancer type. We fitted univariable and multivariable cause-specific accelerated failure time (AFT) Weibull models to estimate cause-specific benefit. Cumulative incidence functions and cause-specific benefits were estimated by year of advanced or metastatic disease diagnosis, SES, race and ethnicity, insurance status, practice type, and sex. The multivariable analysis included all the aforementioned factors. We also examined the potential interaction between SES and insurance status and performed subgroup analyses if a statistically significant interaction effect was detected. In this model, the time ratio (TR) and its 95% CI are estimated. The TR is conceptually similar to the hazard ratio (HR) estimated in the Cox proportional hazards model. When the TR is less than 1, it indicates that an increase in the covariate or not belonging to the reference group is associated with a shorter time to NGS, meaning patients are more likely to undergo NGS sooner. Conversely, when the TR is more than 1, it suggests that an increase in the covariate or not belonging to the reference group is associated with a delay in the time to NGS, meaning patients are more likely to undergo testing later. Potential multicollinearity among covariates was assessed using variance inflation factors (VIFs).^24^ A significance level of .05 was used to determine the statistical significance. All the analysis was done using R version 4.3.2 (R Project for Statistical Computing). Data were analyzed from April 2024 to December 2025.

## Results

### Baseline Characteristics

Overall, 63 294 patients with advanced or metastatic cancer were eligible and included in our study (eFigure 1 in Supplement 1). Of these, 1703 (2.7%) were non-Hispanic Asian, 6551 (10.0%) non-Hispanic Black, 3772 (6.0%) Hispanic, 38 318 (61.0%) non-Hispanic White, 5867 (9.3%) other, and 7083 (11.0%) unknown. The median (IQR) age was 68 (60-76) years and 33 975 patients (53.7%) were female. Among the total 63 294 patients, 12 085 (19.1%) had mBC, 4341 (6.9%) had mPC, 27 050 (42.7%) had aNSCLC, 13 648 (21.6%) had mCRC, and 6170 (9.7%) had mPanC (Table 1).

**Table 1.** Demographics and Patient Characteristics by Cancer Type

Variable	Patients, No. (%)				
	mBC (n = 12 085)	mPC (n = 4341)	aNSCLC (n = 27 050)	mCRC (n = 13 648)	mPanC (n = 6170)
Age at advanced or metastatic diagnosis, median (IQR), y	64 (54-73)	74 (67-81)	70 (62-76)	64 (54-73)	69 (62-75)
Sex					
Female	11 940 (98.8)	NA	13 244 (49.0)	5937 (43.5)	2854 (46.3)
Male	145 (1.2)	4341 (100.0)	13 806 (51.0)	7711 (56.5)	3316 (53.7)
NGS testing rates					
Testing before advanced or metastatic disease diagnosis	695 (5.8)	231 (5.3)	1230 (4.5)	558 (4.1)	0
Testing after advanced or metastatic disease diagnosis	4175 (34.5)	1931 (44.5)	16 599 (61.4)	8584 (62.9)	3162 (51.2)
No testing	7215 (59.7)	2179 (50.2)	9221 (34.1)	4506 (33.0)	3008 (48.8)
No. of therapies received before NGS testing^a^					
0	734 (17.6)	597 (30.9)	10 763 (64.8)	3376 (39.3)	896 (28.3)
1	2150 (51.5)	800 (41.4)	5088 (30.6)	4354 (50.7)	1946 (61.6)
2	760 (18.2)	316 (16.4)	564 (3.4)	607 (7.1)	256 (8.1)
3	304 (7.3)	154 (8.0)	142 (0.9)	161 (1.9)	55 (1.7)
4	138 (3.3)	37 (1.9)	30 (0.2)	56 (0.7)	7 (0.2)
≥5	89 (2.1)	27 (1.4)	12 (0.1)	30 (0.3)	2 (0.1)
Unknown	7910	2410	10 451	5064	3008
Race and ethnicity					
Asian non-Hispanic	268 (2.2)	54 (1.2)	831 (3.1)	423 (3.1)	127 (2.1)
Black non-Hispanic	1490 (12.3)	459 (10.6)	2530 (9.4)	1521 (11.1)	551 (8.9)
Hispanic/Latino	992 (8.2)	255 (5.9)	988 (3.6)	1184 (8.7)	353 (5.7)
White non-Hispanic	7128 (59.1)	2466 (56.8)	17 350 (64.1)	7735 (56.7)	3639 (59.0)
Other^b^	994 (8.2)	634 (14.6)	2266 (8.4)	1191 (8.7)	782 (12.7)
Unknown	1213 (10.0)	473 (10.9)	3085 (11.4)	1594 (11.7)	718 (11.6)
Socioeconomic status					
5 (Highest)	2285 (18.9)	821 (18.9)	4491 (16.6)	2314 (16.9)	1223 (19.8)
4	2533 (20.9)	932 (21.4)	5493 (20.3)	2689 (19.7)	1408 (22.8)
3	2248 (18.6)	832 (19.2)	5332 (19.7)	2483 (18.2)	1157 (18.8)
2	1990 (16.5)	737 (17.0)	5031 (18.6)	2501 (18.3)	997 (16.2)
1 (Lowest)	1944 (16.1)	626 (14.4)	4471 (16.5)	2422 (17.7)	834 (13.5)
Unknown	1085 (9.0)	393 (9.1)	2232 (8.3)	1239 (9.1)	551 (8.9)
Region					
Midwest	1349 (11.2)	452 (10.5)	3307 (12.2)	1501 (11.0)	628 (10.2)
Northeast	1660 (13.7)	629 (14.5)	3969 (14.7)	1874 (13.7)	759 (12.3)
South	4369 (36.1)	1884 (43.4)	10 742 (39.7)	5477 (40.1)	2670 (43.2)
West	1700 (14.1)	631 (14.5)	3351 (12.4)	2233 (16.4)	744 (12.1)
Unknown	3007 (24.9)	745 (17.2)	5681 (21.0)	2563 (18.8)	1369 (22.2)
Insurance					
Commercial health plan	7375 (61.0)	2348 (54.1)	15 449 (57.2)	7674 (56.2)	3995 (64.8)
Medicare or other government program	1249 (10.3)	706 (16.2)	3853 (14.2)	1376 (10.1)	768 (12.4)
Medicaid	249 (2.1)	59 (1.4)	439 (1.6)	280 (2.1)	91 (1.5)
Others	566 (4.7)	216 (5.0)	1079 (4.0)	660 (4.8)	225 (3.6)
Unknown	2646 (21.9)	1012 (23.3)	6230 (23.0)	3658 (26.8)	1091 (17.7)
Practice type					
Community	9615 (79.6)	3789 (87.3)	22 022 (81.4)	11 680 (85.6)	5094 (82.6)
Academic	2470 (20.4)	552 (12.7)	5028 (18.6)	1968 (14.4)	1076 (17.4)

Abbreviations: aNSCLC, advanced non–small cell lung cancer; mBC, metastatic breast cancer; mCRC, metastatic colorectal cancer; mPanC, metastatic pancreatic cancer; mPC, metastatic prostate cancer; NA, not applicable; NGS, next-generation sequencing.

^a^ Percentages were calculated based on the number of patients who received NGS testing.

^b^ Alaska Native, American Indian, Native Hawaiian, other Pacific Islander, or multiracial.

In the mBC cohort, the median (IQR) age was 64 (54-73) years and 11 940 patients (98.8%) were female. A total of 268 patients (2.2%) were non-Hispanic Asian, 1490 (12.3%) non-Hispanic Black, 992 (8.2%) Hispanic, 7128 (59.1%) non-Hispanic White, 994 (8.2%) other, and 1213 (10.0%) unknown. A total of 4175 (34.5%) received NGS after mBC diagnosis.

In the mPC cohort, the median (IQR) age was 74 (67-81) years and 4341 patients (100%) were male. A total of 54 patients (1.2%) were non-Hispanic Asian, 459 (10.6%) non-Hispanic Black, 255 (5.9%) Hispanic, 2466 (56.8%) non-Hispanic White, 634 (14.6%) other, and 473 (10.9%) unknown. A total of 1931 (44.5%) received NGS after mPC diagnosis.

In the aNSCLC cohort, the median (IQR) age was 70 (62-76) years and 13 806 patients (51.0%) were male. Overall, 831 patients (3.1%) were non-Hispanic Asian, 2530 (9.4%) non-Hispanic Black, 988 (3.6%) Hispanic, 17 350 (64.1%) non-Hispanic White, 2266 (8.4%) other, and 3085 (11.4%) unknown. A total of 16 599 (61.4%) received NGS after aNSCLC diagnosis.

In the mCRC cohort, the median (IQR) age was 64 (54-73) years, and 7711 (56.5%) were male. Overall, 423 patients (3.1%) were non-Hispanic Asian, 1521 (11.1%) non-Hispanic Black, 1184 (8.7%) Hispanic, 7735 (56.7%) non-Hispanic White, 1191 (8.7%) other, and 1594 (11.7%) unknown. A total of 8584 (62.9%) received NGS after mCRC diagnosis.

In the mPanC cohort, the median (IQR) age was 69 (62-75) years, and 3316 (53.7%) were male. Overall, 127 patients (2.1%) were non-Hispanic Asian, 551 (8.9%) non-Hispanic Black, 353 (5.7%) Hispanic, 3639 (59.0%) non-Hispanic White, 782 (12.7%) other, and 718 (11.6%) unknown. A total of 3162 (51.2%) received NGS after mPanC diagnosis.

### NGS Testing by Tumor Type

In the mBC cohort, median time to NGS was 2.9 (95% CI, 2.7-3.1) months. The cumulative incidence of NGS was 26.2% (95% CI, 25.4%-27.0%) at 1 year after mBC diagnosis and 44.0% (95% CI, 42.8%-45.3%) at the data cutoff. For patients diagnosed in 2018, cumulative incidence was 13.0% (95% CI, 11.8%-14.3%) at 1 year and 35.2% (95% CI, 33.1%-37.3%) at the data cutoff. For those diagnosed in 2022, cumulative incidence was 39.3% (95% CI, 36.3%-42.2%) at 1 year and 44.9% (95% CI, 35.7%-53.8%) at the data cutoff.

In the mPC cohort, median time to NGS was 11.0 (95% CI, 9.8-11.0) months. The cumulative incidence of NGS was 24.6% (95% CI, 23.3%-25.9%) at 1 year after mPC diagnosis and 54.4% (95% CI, 52.3%-56.4%) at the data cutoff. For patients diagnosed in 2018, cumulative incidence was 10.3% (95% CI, 8.6%-12.1%) at 1 year and 44.1% (95% CI, 40.8%-47.4) at the data cutoff. For those diagnosed in 2022, cumulative incidence was 37.1% (95% CI, 30.5%-43.6%) at 1 year and 37.1% (95% CI, 30.5%-43.6%) at the data cutoff.

In the aNSCLC cohort, median time to NGS was 0.96 (95% CI, 0.93-0.96) months. The cumulative incidence of NGS was 58.3% (95% CI, 57.7%-58.9%) at 1 year after aNSCLC diagnosis and 64.7% (95% CI, 64.0%-65.4%) at the data cutoff. For patients diagnosed in 2018, cumulative incidence was 41.9% (95% CI, 40.7%-43.1%) at 1 year and 50.1% (95% CI, 48.7%-51.5%) at the data cutoff. For those diagnosed in 2022, cumulative incidence was 74.5% (95% CI, 72.8%-76.0%) at 1 year and 75.7% (95% CI, 72.7%-78.5%) at the data cutoff.

In the mCRC cohort, median time to NGS was 1.5 (95% CI, 1.5-1.6) months. The cumulative incidence of NGS was 56.3% (95% CI, 55.5%-57.2%) at 1 year after mCRC diagnosis and 68.1% (95% CI, 67.2%-69.0%) at the data cutoff. For patients diagnosed in 2018, cumulative incidence was 41.2% (95% CI, 39.5%-43.0%) at 1 year and 57.2% (95% CI, 55.3%-59.0%) at the data cutoff. For those diagnosed in 2022, cumulative incidence was 78.1% (95% CI, 75.7%-80.4%) at 1 year and 79.1% (95% CI, 76.0%-81.8%) at the data cutoff.

In the mPanC cohort, median time to NGS was 1.3 (95% CI, 1.2-1.3) months. The cumulative incidence of NGS was 50.3% (95% CI, 49.0%-51.6%) at 1 year after mPanC diagnosis and 54.5% (95% CI, 53.1%-55.8%) at the data cutoff. For patients diagnosed in 2018, cumulative incidence was 29.8% (95% CI, 27.3%-32.3%) at 1 year and 36.0% (95% CI, 33.3%-38.7%) at the data cutoff. For those diagnosed in 2022, cumulative incidence was 62.2% (95% CI, 58.6%-65.6%) at 1 year and 62.2% (95% CI, 58.6%-65.6%) at the data cutoff.

**Figure 1.**
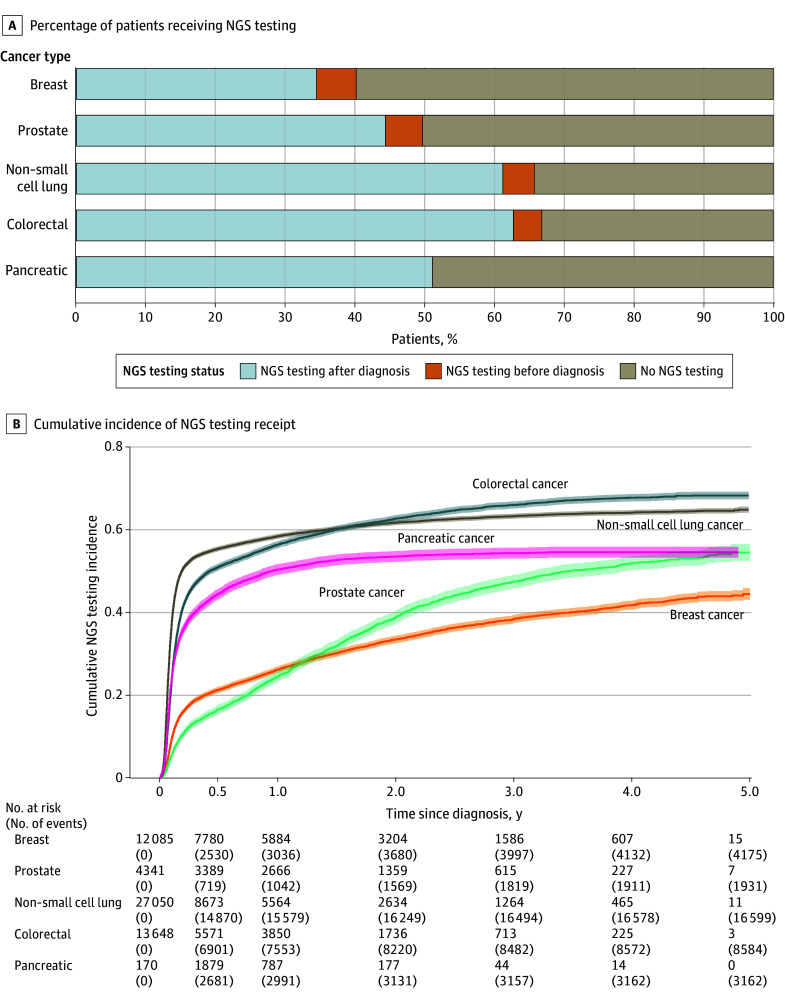
Bar Chart and Survival Curve Showing Cumulative Incidence by Cancer Type After the Onset of Advanced or Metastatic Disease Diagnosis NGS indicates next-generation sequencing. Shading represents 95% CIs.

Figure 1 shows cumulative incidence of testing after advanced or metastatic disease diagnosis by cancer type. eFigure 2 in Supplement 1 shows trends in cumulative incidence of NGS by year of advanced or metastatic disease diagnosis and cancer type at 1 year and at the data cutoff. eFigure 3 in Supplement 1 shows the CIFs of NGS and death by cancer type following metastatic or advanced disease diagnosis.

**Figure 2.**
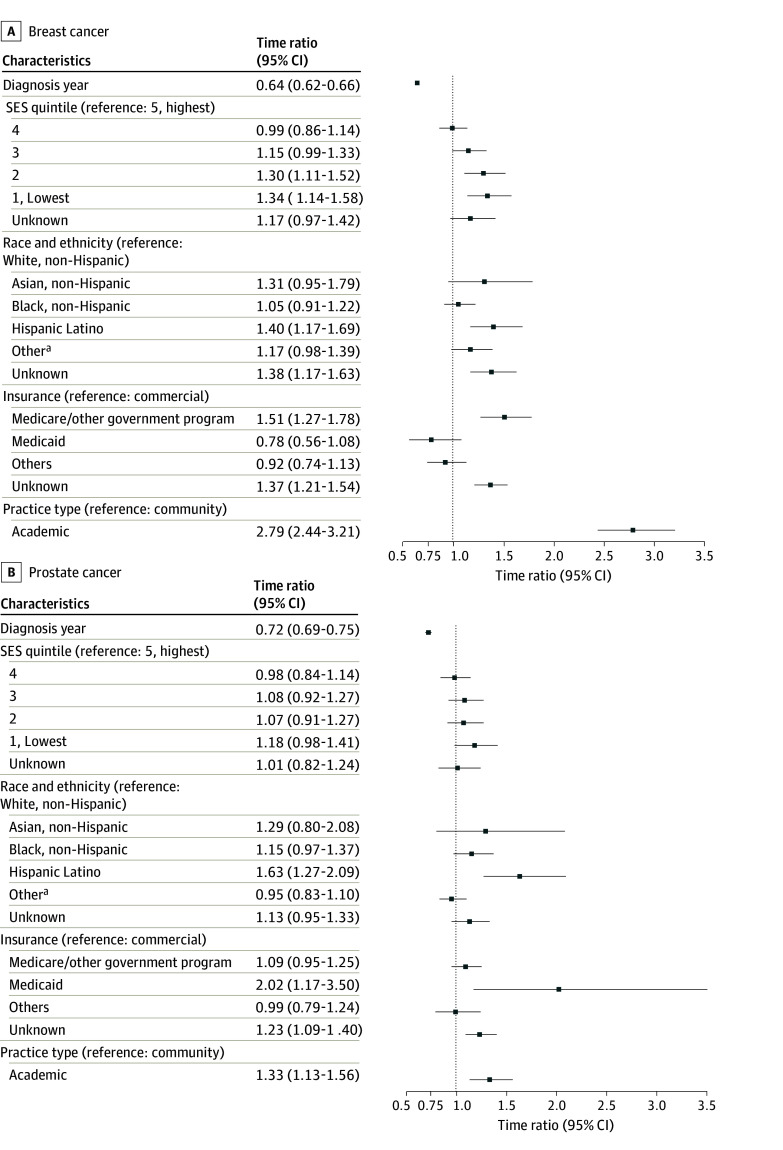
Forest Plots Showing the Multivariable Cause-Specific Weibull Accelerated Failure Time Model SES indicates socioeconomic status. ^a^Other race and ethnicity includes Alaska Native, American Indian, Native Hawaiian, other Pacific Islander, or multiracial.

**Figure 3.**
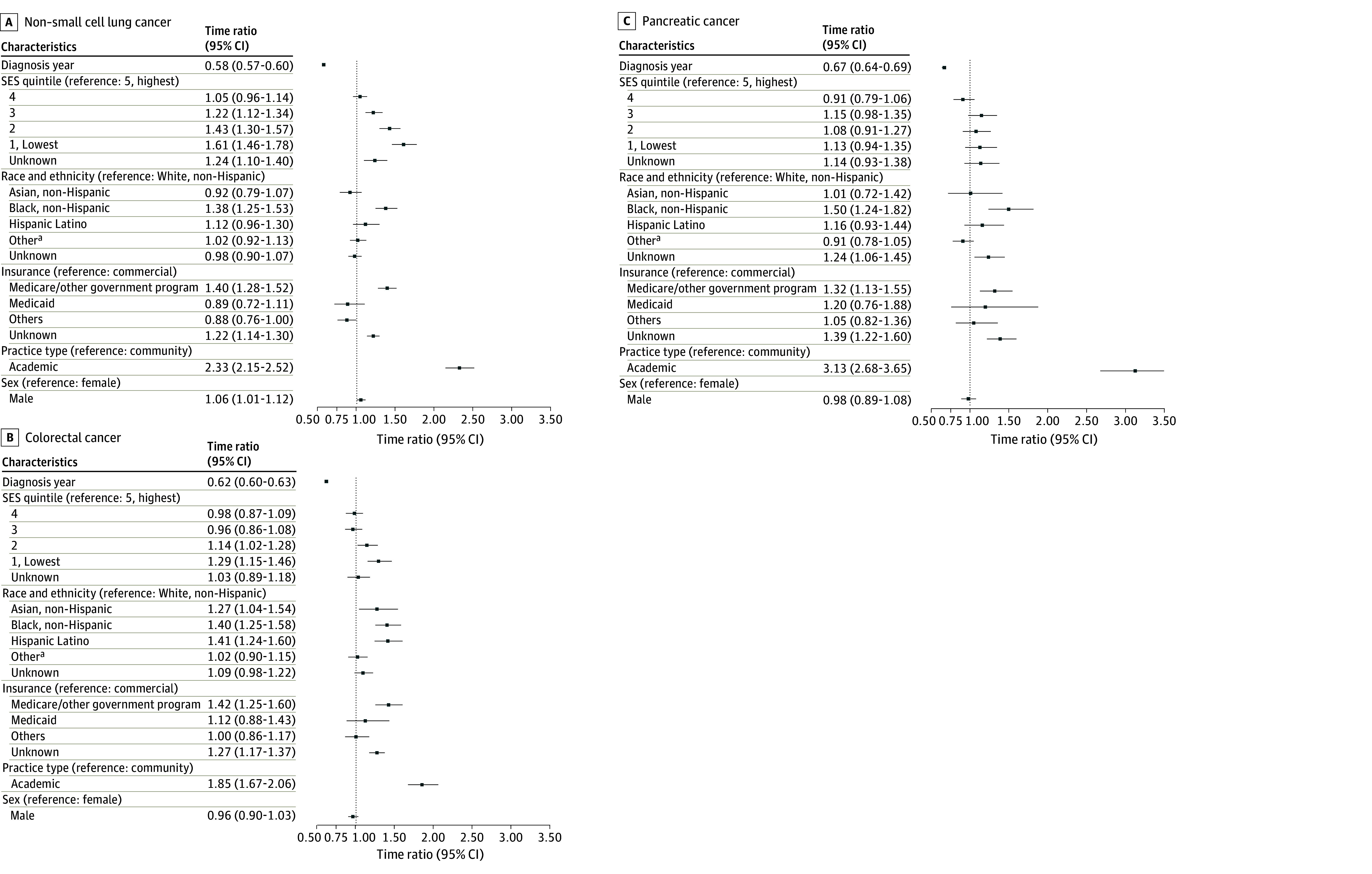
Forest Plots Showing the Multivariable Cause-Specific Weibull Accelerated Failure Time Model SES indicates socioeconomic status. ^a^Other race and ethnicity includes Alaska Native, American Indian, Native Hawaiian, other Pacific Islander, or multiracial.

The results from the multivariable cause-specific Weibull accelerated failure time (AFT) model are presented in Figures 2 and 3. The results of the multivariable and univariable cause-specific Weibull AFT analyses are shown in eTable 1 in Supplement 1, respectively. No evidence of severe multicollinearity among covariates was observed, with all VIF less than 5. In interaction analyses, a statistically significant interaction between SES and insurance type was observed in mPC (χ^2^_20_ = 45.58; *P* < .001) and aNSCLC (χ^2^_20_ = 31.56; *P* = .048) (eTables 2, 3, 4, and 5 in Supplement 1).

### Disparities in Patients With mBC

Between 2018 and 2022, time to NGS in patients with mBC significantly decreased by year of metastatic diagnosis (TR, 0.64; 95% CI, 0.62-0.66; *P* < .001). Compared with patients with the highest SES, those with low SES experienced a significantly longer time to NGS (1: TR, 1.3; 95% CI, 1.1-1.6; *P* < .001; 2: TR, 1.3; 95% CI, 1.1-1.5; *P* = .001). Hispanic patients (TR, 1.4; 95% CI, 1.2-1.7; *P* < .001), those covered by Medicare or other government programs (TR, 1.5; 95% CI, 1.3-1.8; *P* < .001), and those treated in academic practice (TR, 2.8; 95% CI, 2.4-3.2; *P* < .001) had a significantly longer time to NGS.

### Disparities in Patients With mPC

In patients with mPC, time to NGS significantly decreased by year of metastatic diagnosis (TR, 0.72; 95% CI, 0.69-0.75; *P* < .001). Hispanic patients (TR, 1.6; 95% CI, 1.3-2.1; *P* < .001), those covered by Medicaid (TR, 2.0; 95% CI, 1.2-3.5; *P* = .01), and those treated in academic practice (TR, 1.3; 95% CI, 1.1-1.6; *P* < .001) had a significantly longer time to NGS.

Subgroup analyses stratified by insurance type and SES demonstrated significant effect modification between SES and insurance type on time to NGS (eTables 2 and 3 in Supplement 1). Among commercially insured and uninsured or unknown patients, lower SES was generally associated with longer time to NGS, whereas this gradient was attenuated among Medicare beneficiaries. Medicare coverage was associated with longer time to NGS primarily among patients in the highest SES group, and Medicaid coverage was associated with longer time to NGS predominantly in middle SES strata.

### Disparities in Patients With aNSCLC

In patients with aNSCLC, time to NGS significantly decreased by year of advanced disease diagnosis (TR, 0.58; 95% CI, 0.57-0.60; *P* < .001). Compared with patients with the highest SES, those with low SES experienced a significantly longer time to NGS (1: TR, 1.6; 95% CI, 1.5-1.8; *P* < .001; 2: TR 1.4; 95% CI, 1.3-1.6; *P* < .001; 3: TR, 1.2; 95% CI, 1.1-1.3; *P* < .001). Non-Hispanic Black patients (TR, 1.4; 95% CI, 1.2-1.5; *P* < .001), those covered by Medicare or other government programs (TR, 1.4; 95% CI, 1.3-1.5; *P* < .001), and those treated in academic practice (TR, 2.3; 95% CI, 2.2-2.5; *P* < .001) had a significantly longer time to NGS. Compared with female patients, male patients experienced a significantly longer time to NGS after aNSCLC diagnosis (TR, 1.1; 95% CI, 1.0-1.1; *P* = .03).

Subgroup analyses stratified by insurance and SES demonstrated effect modification between SES and insurance on time to NGS (eTables 4 and 5 in Supplement 1). When stratified by insurance, lower SES was associated with longer time to NGS among commercially insured patients, but this gradient was attenuated among Medicare beneficiaries. When stratified by SES, Medicare coverage was associated with longer time to NGS across most SES strata, while Medicaid coverage was not consistently associated with delayed testing across SES groups.

### Disparities in Patients With mCRC

In patients with mCRC, time to NGS significantly decreased by year of metastatic disease diagnosis (TR, 0.62; 95% CI, 0.60-0.63; *P* < .001). Compared with patients with the highest SES, those with low SES experienced a significantly longer time to NGS (1: TR, 1.3; 95% CI, 1.1-1.5; *P* < .001; 2: TR, 1.1; 95% CI, 1.0-1.3; *P* = .03). Non-Hispanic Black (TR, 1.4; 95% CI, 1.2-1.6; *P* < .001) and Hispanic (TR, 1.4; 95% CI, 1.2-1.6; *P* < .001) patients, those covered by Medicare or other government programs (TR, 1.4; 95% CI, 1.3-1.6; *P* < .001), and those treated in academic practice (TR, 1.9; 95% CI, 1.7-2.1; *P* < .001) had a significantly longer time to NGS.

### Disparities in Patients With mPanC

In patients with mPanC, time to NGS significantly decreased by year of metastatic disease diagnosis (TR, 0.67; 95% CI, 0.64-0.69; *P* < .001). Non-Hispanic Black (TR, 1.5; 95% CI, 1.2-1.8; *P* < .001) patients, those covered by Medicare or other government programs (TR, 1.3; 95% CI, 1.1-1.5; *P* < .001), and those treated in academic practice (TR, 3.1; 95% CI, 2.7-3.7; *P* < .001) had a significantly longer time to NGS.

## Discussion

Our study is one of the largest to assess NGS utilization and its association with social determinants of health with access to genomic profiling in a large nationwide patient-level dataset including the 5 most common advanced cancers in the US. While the cumulative incidence of NGS at 1 year after advanced or metastatic disease diagnosis improved in patients diagnosed in 2022 compared with 2018 across all cancer types, a sizeable number of patients still did not undergo genomic testing. Additionally, patients with low SES, non-Hispanic Black or Hispanic patients, those covered by Medicaid, Medicare, or other government programs, and patients treated in academic practice were significantly less likely to be tested in some of these cancers, compared with patients with high SES, non-Hispanic White patients, those covered by a commercial health plan, or those treated in community practice, respectively, highlighting the need to improve access to standard-of-care diagnostic modalities.

Recently, precision oncology has defined a new era in cancer treatment, enabling clinicians to tailor medical care based on the unique clinicogenomic features of a patient’s tumor, thereby allowing for more effective and less toxic treatment strategies. Several targeted therapies approved based on genomic profiling have led to dramatic improvement in survival outcomes of patients with cancer. For instance, in the phase 3 FLAURA trial, patients with *EGFR*-alteration aNSCLC receiving osimertinib (EGFR-TKI) had a 54% reduction in the risk of progression or death compared with those treated with a standard EGFR-TKI.^25^ Similarly, in prostate cancer, patients with mCRPC harboring HRR alterations had a 45% reduction in the risk of death when receiving the combination of talazoparib and enzalutamide compared with enzalutamide alone.^26^ However, multiple barriers continue to hinder the implementation of NGS in clinical settings, which could help explain the low use of NGS encountered in our study.^27^ These include physician- and patient-related challenges, financial constraints, and infrastructure limitations.^27^ Addressing these obstacles through health care policies that enhance access to genomic testing, increase awareness, and promote education on level-1 evidence is crucial for optimizing the benefits of precision oncology.

Our findings suggest that Hispanic patients were significantly less likely to be tested in mBC and mPC, and non-Hispanic Black patients were less likely to receive NGS in aNSCLC, mCRC, and mPanC. Evidence from previous US-based studies on racial and ethnic disparities in genomic testing among patients with cancer has provided mixed results. In an institutional retrospective study between 2014 and 2019 that included 3461 patients with mBC, mCRC, mPC, and aNSCLC, 44.5% underwent genomic profiling.^20^ Additionally, non-Hispanic Black patients were significantly less likely to be tested in the mBC and mPC cohorts, with no differences found in the aNSCLC and mPanC cohorts. In another nationwide study that used the Medicare Standard Analytic Files database, only 1.8% of patients received NGS across different tumor types between 2015 and 2020, and non-Hispanic Black and Hispanic patients were less likely to undergo NGS.^19^ The plausible causes for these racial and ethnic disparities include lower rates of referrals for genomic testing, reduced attendance at genetic counseling appointments, limited awareness and education about NGS, and historical mistrust of the health care system among minoritized groups.^18,19,20,27^

Another pertinent finding in our study is that patients with low SES were less likely to be tested in mBC, aNSCLC, and mCRC, and patients covered by Medicaid or Medicare experienced barriers to testing across all tumor types. A range of interconnected factors may account for the lower likelihood of these patients undergoing tumor genomic profiling. Limited access to health care in their communities often restricts opportunities for advanced diagnostic testing.^28^ Moreover, the high cost of NGS,^29^ combined with inconsistent insurance coverage, can further discourage its use.^18^ In fact, in 2018, Medicare issued a National Coverage Determination (NCD) memorandum designating NGS as an essential diagnostic tool for patients with advanced or metastatic cancer, which could enhance access to NGS for individuals with low SES.^30^ The release of this memorandum was followed by an increase in the rates of NGS in different cancer types across all insurance plans.^31^ However, increases in NGS following the 2018 NCD were not uniform across patient populations, with smaller relative gains observed among non-Hispanic Black and Hispanic patients, indicating persistent disparities in access to genomic testing.^31^

To our knowledge, this is one of the largest studies to date examining the use and disparities in patients with the most common advanced cancers in the US. Using a patient-level database spanning 5 years, we analyzed changes in NGS utilization among the 5 most common advanced cancers in the US. Our findings highlight the underrepresentation of certain patient demographics in tumor genomic profiling, revealing disparities in access to standard-of-care diagnostic modalities. These results emphasize the need for health care policies to mitigate these gaps.

### Limitations

The limitations of our study include retrospective design, data missingness in certain patient exposures due to reliance on EHRs, and misclassification bias. The study focused on multigene testing performed using NGS-based platforms and did not capture single-gene, hotspot, or other targeted biomarker assays, which may be more commonly used in certain settings, including academic centers. The study also does not distinguish between somatic and germline NGS. All exposures were assessed at baseline (ie, date of advanced or metastatic disease diagnosis), and we were unable to account for potential biases, such as access to genetic counseling or genomic testing, or changes over time in patient characteristics. Differences in the availability and line of therapy of targeted treatments across cancer types during the study period may also have influenced the timing of NGS, as actionable alterations in some cancers historically guided later-line rather than first-line treatment decisions. Additionally, line of therapy was not modeled as a competing event, and patients who proceeded to later lines of treatment may have had greater opportunity to undergo NGS. Additional unmeasured confounding factors that may have influenced testing include physician specialty, patient preferences, clinician awareness of guidelines, and tumor-related factors.

## Conclusions

In this cohort study of patients with advanced or metastatic cancers in the US, although the 1-year cumulative incidence of NGS improved over time across all cancer types, many patients with cancer did not undergo NGS. Social determinants of health, including race and ethnicity, SES, insurance coverage, practice type, and sex, might have influenced access to NGS. These benchmark data provide valuable insights into current utilization and disparities in access to NGS. These results have the potential to inform health care policies and educational efforts aimed at bridging these gaps and addressing the underutilization of NGS in the most common advanced cancers in the US.

## Notes

Supplement 1. eTable 1. Univariable Cause-Specific Weibull AFT Model in Different Cancer Types by Advanced or Metastatic Diagnosis Year, Socioeconomic Status, Race-Ethnicity, Insurance Status, and Sex eTable 2. Subgroup Analysis of Prostate Cancer Stratified by Insurance Type eTable 3. Subgroup Analysis of Prostate Cancer Stratified by Socioeconomic Status eTable 4. Subgroup Analysis of Non–Small Cell Lung Cancer Stratified by Insurance Type eTable 5. Subgroup Analysis of Non–Small Cell Lung Cancer Stratified by Socioeconomic Status eFigure 1. Study Cohort eFigure 2. Trends in the Cumulative Incidence of NGS Testing by Cancer Type and Year of Advanced or Metastatic Disease Diagnosis After 1 Year of the Advanced or Metastatic Disease Diagnosis and at the Data Cutoff Date eFigure 3. Cumulative Incidence Functions of NGS Testing and Death by Cancer Type Following Metastatic or Advanced Diagnosis

Supplement 2. Data Sharing Statement
