# Error in Abstract and Conclusions

**DOI:** 10.1001/jamanetworkopen.2026.16328

**Published:** 2026-05-07

In the Original Investigation titled “Trends and Disparities in the Use of Next-Generation Sequencing in Patients With Cancer in the United States,”^1^ published April 7, 2026, there were errors in the Abstract and Conclusions. The text should have stated that many patients did not undergo tumor genomic testing rather than stating most patients did not undergo tumor genomic testing.^1^
