# Supplementary material for: Trends and Disparities in the Use of Next-Generation Sequencing in Patients With Cancer in the United States
Source: JAMA Netw Open. 2026 Apr 7;9(4):e265585. doi: 10.1001/jamanetworkopen.2026.5585 (PMC13058762; doi:10.1001/jamanetworkopen.2026.5585)
Supplement: Supplement 1. — eTable 1. Univariable Cause-Specific Weibull AFT Model in Different Cancer Types by Advanced or Metastatic Diagnosis Year, Socioeconomic Status, Race-Ethnicity, Insurance Status, and Sex eTable 2. Subgroup Analysis of Prostate Cancer Stratified by Insurance Type eTable 3. Subgroup Analysis of Prostate Cancer Stratified by Socioeconomic Status eTable 4. Subgroup Analysis of Non–Small Cell Lung Cancer Stratified by Insurance Type eTable 5. Subgroup Analysis of Non–Small Cell Lung Cancer Stratified by Socioeconomic Status eFigure 1. Study Cohort eFigure 2. Trends in the Cumulative Incidence of NGS Testing by Cancer Type and Year of Advanced or Metastatic Disease Diagnosis After 1 Year of the Advanced or Metastatic Disease Diagnosis and at the Data Cutoff Date eFigure 3. Cumulative Incidence Functions of NGS Testing and Death by Cancer Type Following Metastatic or Advanced Diagnosis [file jamanetwopen-e265585-s001.pdf]

Supplemental Online Content

Chehade CH, Ozay ZI, Jo Y, et al. Trends and disparities in the use of next-generation sequencing for cancer. *JAMA Netw Open*. 2026;9(4):e265585. doi:10.1001/jamanetworkopen.2026.5585

eTable 1. Univariable Cause-Specific Weibull AFT Model in Different Cancer Types by Advanced or Metastatic Diagnosis Year, Socioeconomic Status, Race-Ethnicity, Insurance Status, and Sex
eTable 2. Subgroup Analysis of Prostate Cancer Stratified by Insurance Type
eTable 3. Subgroup Analysis of Prostate Cancer Stratified by Socioeconomic Status
eTable 4. Subgroup Analysis of Non–Small Cell Lung Cancer Stratified by Insurance Type
eTable 5. Subgroup Analysis of Non–Small Cell Lung Cancer Stratified by Socioeconomic Status
eFigure 1. Study Cohort
eFigure 2. Cumulative Incidence Functions of NGS Testing and Death by Cancer Type Following Metastatic or Advanced Diagnosis

This supplemental material has been provided by the authors to give readers additional information about their work.

© 2026 Chehade CH et al. *JAMA Network Open*.

eTable 1. Univariable Cause-Specific Weibull AFT Model in Different Cancer Types by Advanced or Metastatic Diagnosis Year, Socioeconomic Status, Race-Ethnicity, Insurance Status, and Sex

	mBC	mPC	aNSCLC	mCRC	mPanC
**Variable**	**TR (95% CI, p)**				
**Advanced/metastatic diagnosis year**	0.64 (0.62-0.66, <0.001)	0.72 (0.69-0.75, <0.001)	0.58 (0.57-0.59, <0.001)	0.61 (0.59-0.63, < 0.001)	0.67 (0.64-0.69, < 0.001)
**Socioeconomic status**					
5 (Highest)	Ref	Ref	Ref	Ref	Ref
4	0.93 (0.80-1.1, 0.4)	0.96 (0.81-1.1, 0.6)	1.0 (0.94-1.1, 0.5)	0.96 (0.85-1.1, 0.5)	0.81 (0.69-0.95, 0.009)
3	1.0 (0.87-1.2, 0.8)	1.0 (0.87-1.2, 0.7)	1.1 (1.0-1.2, 0.011)	0.94 (0.83-1.1, 0.3)	1.0 (0.85-1.2, 0.9)
2	1.1 (0.93-1.3, 0.2)	1.1 (0.88-1.3, 0.6)	1.3 (1.2-1.4, <0.001)	1.1 (1.0-1.3, 0.035)	0.92 (0.77-1.1, 0.3)
1 (Lowest)	1.2 (1.0-1.5, 0.023)	1.2 (1.0-1.5, 0.027)	1.5 (1.4-1.7, <0.001)	1.4 (1.2-1.5, <0.001)	1.0 (0.84-1.2, 0.9)
Unknown	1.2 (0.94-1.4, 0.2)	1.0 (0.82-1.3, 0.9)	1.3 (1.1-1.5, <0.001)	1.1 (0.94-1.3, 0.2)	0.97 (0.79-1.2, 0.8)
**Race / Ethnicity**					
White non-Hispanic	Ref	Ref	Ref	Ref	Ref
Asian non-Hispanic	1.2 (0.82-1.6, 0.4)	1.4 (0.85-2.4, 0.2)	0.79 (0.67-0.93, 0.006)	1.2 (0.94-1.4, 0.2)	0.92 (0.64-1.3, 0.7)
Black non-Hispanic	1.1 (0.91-1.3, 0.4)	1.3 (1.0-1.5, 0.016)	1.5 (1.4-1.7, <0.001)	1.5 (1.3-1.7, <0.001)	1.4 (1.2-1.8, <0.001)
Hispanic/Latino	1.4 (1.1-1.7, 0.001)	1.7 (1.3-2.3, <0.001)	1.1 (0.97-1.3, 0.11)	1.5 (1.3-1.7, <0.001)	1.1 (0.84-1.3, 0.6)
Other[a]	1.1 (0.87-1.3, 0.6)	0.97 (0.83-1.1, 0.6)	0.91 (0.82-1.0, 0.08)	0.95 (0.83-1.1, 0.4)	0.74 (0.63-0.86, <0.001)
Unknown	1.1 (0.93-1.3, 0.2)	1.1 (0.88-1.3, 0.6)	0.76 (0.69-0.84, <0.001)	0.86 (0.76-0.96, 0.009)	0.87 (0.73-1.0, 0.09)
**Insurance**					
Commercial health plan	Ref	Ref	Ref	Ref	Ref
Medicare / other government program	1.7 (1.4-2.1, < 0.001)	1.2 (1.1-1.4, 0.009)	1.7 (1.5-1.8, <0.001)	1.6 (1.4-1.9, <0.001)	1.5 (1.3-1.8, <0.001)
Medicaid	0.92 (0.65-1.3, 0.7)	2.3 (1.3-4.1, 0.007)	1.0 (0.83-1.3, 0.7)	1.3 (0.99-1.7, 0.062)	1.8 (1.1-2.9, 0.018)

© 2026 Chehade CH et al. *JAMA Network Open*.

Others	1.1 (0.83-1.3, 0.7)	1.0 (0.80-1.3, 0.8)	0.87 (0.75-1.0, 0.061)	1.0 (0.84-1.2, 0.9)	1.1 (0.80-1.4, 0.7)
Unknown	1.8 (1.6-2.1, <0.001)	1.5 (1.3-1.7, <0.001)	1.6 (1.5-1.7, <0.001)	1.6 (1.5-1.8, <0.001)	1.7 (1.5-2.0, <0.001)
**Practice Type**					
Community	Ref	Ref	Ref	Ref	Ref
Academic	2.7 (2.3-3.1, <0.001)	1.3 (1.1-1.5, 0.002)	2.3 (2.1-2.4, <0.001)	1.9 (1.7-2.1, <0.001)	3.1 (2.7-3.6, <0.001)
**Sex**					
Female	Ref	Ref	Ref	Ref	Ref
Male	NA	NA	1.1 (1.0-1.1, 0.03)	0.95 (0.89-1.0, 0.2)	0.99 (0.89-1.1, 0.8)

[a] Alaska Native, American Indian, Native Hawaiian, other Pacific Islander or multiracial.

Abbreviations: AFT, accelerated failure time; aNSCLC, advanced non-small cell lung cancer; CI, confidence interval; mBC, metastatic breast cancer; mCRC, metastatic colorectal cancer; mPanC, metastatic pancreatic cancer; mPC, metastatic prostate cancer; NA, not applicable; TR, time ratio.

© 2026 Chehade CH et al. *JAMA Network Open*.

eTable 2. Subgroup Analysis of Prostate Cancer Stratified by Insurance Type

Characteristic	Commercial health plan TR (95% CI)	Medicare/other government program TR (95% CI)	Medicaid TR (95% CI)	Others TR (95% CI)	Unknown TR (95% CI)
**Advanced/metastatic diagnosis year**	0.73 (0.69–0.77)	0.72 (0.66–0.78)	0.69 (0.40–1.20)	0.74 (0.61–0.91)	0.71 (0.65–0.76)
**SES**					
5 (Highest)	Ref	Ref	Ref	Ref	Ref
4	1.1 (0.91–1.4)	0.64 (0.45–0.93)	NA	0.58 (0.28–1.2)	1.1 (0.81–1.4)
3	1.1 (0.91–1.4)	0.64 (0.44–0.93)	NA	1.6 (0.68–3.7)	1.2 (0.90–1.6)
2	1.2 (0.91–1.5)	0.58 (0.39–0.87)	NA	1.8 (0.77–4.4)	1.3 (0.98–1.7)
1 (Lowest)	1.20 (0.90–1.50)	0.72 (0.47–1.10)	NA	2.90 (1.10–7.40)	1.30 (0.96–1.70)
**Race/ethnicity**					
White non-Hispanic	Ref	Ref	Ref	Ref	Ref
Asian non-Hispanic	0.90 (0.43–1.9)	0.96 (0.31–2.9)	NA	1.3 (0.23–7.3)	1.8 (0.85–4.0)
Black non-Hispanic	1.20 (0.89–1.50)	1.30 (0.87–1.90)	2.00 (0.55–7.10)	1.20 (0.50–2.70)	1.10 (0.82–1.40)
Hispanic	1.70 (1.10–2.50)	1.40 (0.72–2.90)	0.68 (0.13–3.70)	0.79 (0.27–2.30)	1.70 (1.20–2.40)
Other	0.95 (0.78–1.2)	1.0 (0.74–1.4)	3.5 (0.61– 20)	0.65 (0.31– 1.3)	0.96 (0.75–1.2)
Unknown	1.2 (0.92–1.5)	0.79 (0.54–1.1)	0.47 (0.12–1.8)	1.6 (0.67–3.8)	1.2 (0.91–1.6)
**Practice type**					
Community	Ref	Ref	Ref	Ref	Ref
Academic	1.40 (1.10–1.80)	1.20 (0.89–1.70)	3.10 (0.40–24.0)	1.10 (0.47–2.80)	1.10 (0.90–1.50)

Abbreviations: CI, confidence interval; HR, hazard ratio; NA, not available; SES, socioeconomic status.

© 2026 Chehade CH et al. *JAMA Network Open*.

eTable 3. Subgroup Analysis of Prostate Cancer Stratified by Socioeconomic Status

Characteristic	SES 5 (Highest) TR (95% CI)	SES 4 TR (95% CI)	SES 3 TR (95% CI)	SES 2 TR (95% CI)	SES 1 (Lowest) TR (95% CI)	SES Unknown TR (95% CI)
**Advanced/metastatic diagnosis year**	0.73 (0.67–0.81)	0.69 (0.64–0.75)	0.74 (0.68–0.81)	0.78 (0.70–0.86)	0.72 (0.64–0.80)	0.67 (0.59–0.76)
**Race/ethnicity**						
White non-Hispanic	Ref	Ref	Ref	Ref	Ref	Ref
Asian non-Hispanic	0.90 (0.39–2.1)	2.1 (0.63–7.1)	4.2 (0.47–38)	1.2 (0.33–4.3)	2.1 (0.48–9.0)	0.99 (0.31–3.2)
Black non-Hispanic	0.96 (0.57–1.6)	1.0 (0.68–1.5)	1.1 (0.73–1.6)	1.4 (0.93–2.1)	1.2 (0.86–1.7)	1.2 (0.68–2.1)
Hispanic	0.85 (0.43–1.70)	2.60 (1.20–5.50)	1.90 (0.97–3.80)	1.00 (0.63–1.60)	1.60 (0.97–2.70)	2.50 (1.20–5.00)
Other	0.89 (0.62–1.3)	0.86 (0.65–1.1)	1.0 (0.74–1.4)	0.89 (0.64–1.2)	1.0 (0.72–1.5)	1.1 (0.70–1.6)
Unknown	1.1 (0.75–1.7)	1.1 (0.77–1.5)	1.3 (0.88–1.9)	1.1 (0.76–1.6)	0.75 (0.49–1.2)	1.7 (0.84–3.6)
**Insurance type**						
Commercial health plan	Ref	Ref	Ref	Ref	Ref	Ref
Medicare/other government program	1.90 (1.30–2.70)	1.00 (0.76–1.30)	1.00 (0.76–1.40)	0.87 (0.63–1.20)	1.20 (0.80–1.70)	0.69 (0.44–1.10)
Medicaid	NA	2.00 (0.45–8.80)	10.0 (1.10–91.0)	0.85 (0.34–2.10)	2.50 (0.76–8.40)	0.56 (0.17–1.80)
Others	0.90 (0.52–1.6)	0.52 (0.34–0.79)	1.3 (0.73–2.4)	1.3 (0.71–2.4)	1.9 (1.0–3.7)	0.77 (0.37–1.6)
Unknown	1.2 (0.89–1.7)	1.1 (0.83–1.4)	1.3 (0.94–1.7)	1.4 (1.1–2.0)	1.4 (1.0–1.9)	1.1 (0.71–1.6)
**Practice type**						
Community	Ref	Ref	Ref	Ref	Ref	Ref
Academic	1.30 (0.90–1.70)	1.30 (0.99–1.80)	1.10 (0.79–1.70)	1.40 (0.88–2.10)	2.90 (1.30–6.40)	0.79 (0.46–1.40)

Abbreviations: CI, confidence interval; HR, hazard ratio; NA, not available; SES, socioeconomic status.

© 2026 Chehade CH et al. *JAMA Network Open*.

eTable 4. Subgroup Analysis of Non–Small Cell Lung Cancer Stratified by Insurance Type

Characteristic	Commercial health plan TR (95% CI)	Medicare/other government program TR (95% CI)	Medicaid TR (95% CI)	Others TR (95% CI)	Unknown TR (95% CI)
**Advanced/metastatic diagnosis year**	0.60 (0.58–0.62)	0.56 (0.53–0.60)	0.59 (0.50–0.70)	0.57 (0.52–0.63)	0.56 (0.53–0.58)
**SES**					
5 (Highest)	Ref	Ref	Ref	Ref	Ref
4	1.0 (0.93–1.2)	0.92 (0.71–1.2)	1.3 (0.52–3.3)	1.4 (0.87–2.1)	1.1 (0.94–1.4)
3	1.2 (1.1–1.4)	1.2 (0.89–1.5)	0.93 (0.37–2.3)	1.0 (0.65–1.5)	1.4 (1.2– 1.7)
2	1.4 (1.2–1.6)	1.3 (1.0–1.7)	1.4 (0.59–3.4)	1.6 (1.0–2.4)	1.6 (1.3–1.9)
1 (Lowest)	1.70 (1.50–1.90)	1.10 (0.83–1.50)	1.90 (0.77–4.60)	2.10 (1.30–3.30)	1.70 (1.40–2.10)
**Race/ethnicity**					
White non-Hispanic	Ref	Ref	Ref	Ref	Ref
Asian non-Hispanic	1.0 (0.85–1.3)	0.46 (0.30–0.72)	0.33 (0.13– 0.83)	0.96 (0.55–1.7)	0.96 (0.70–1.3)
Black non-Hispanic	1.50 (1.30–1.80)	1.40 (1.10–1.90)	0.67 (0.33–1.30)	0.96 (0.61–1.50)	1.20 (0.95–1.40)
Hispanic	1.30 (1.10–1.60)	0.97 (0.59–1.60)	0.97 (0.44–2.10)	1.20 (0.71–1.90)	0.88 (0.66–1.20)
Other	0.99 (0.87–1.1)	1.2 (0.85–1.6)	0.65 (0.29– 1.4)	0.99 (0.66–1.5)	1.1 (0.86–1.3)
Unknown	0.94 (0.84–1.1)	0.89 (0.69–1.1)	1.2 (0.45–3.1)	1.7 (1.1–2.6)	1.0 (0.86–1.2)
**Practice type**					
Community	Ref	Ref	Ref	Ref	Ref
Academic	2.50 (2.20–2.80)	3.10 (2.50–3.90)	0.44 (0.26–0.74)	2.10 (1.40–3.30)	2.00 (1.80–2.40)

Abbreviations: CI, confidence interval; HR, hazard ratio; NA, not available; SES, socioeconomic status.

© 2026 Chehade CH et al. *JAMA Network Open*.

eTable 5. Subgroup Analysis of Non–Small Cell Lung Cancer Stratified by Socioeconomic Status

Characteristic	SES 5 (Highest) TR (95% CI)	SES 4 TR (95% CI)	SES 3 TR (95% CI)	SES 2 TR (95% CI)	SES 1 (Lowest) TR (95% CI)	SES Unknown TR (95% CI)
**Advanced/metastatic diagnosis year**	0.62 (0.59–0.65)	0.58 (0.55–0.61)	0.60 (0.58–0.63)	0.55 (0.53–0.58)	0.57 (0.54–0.60)	0.59 (0.55–0.63)
**Race/ethnicity**						
White non-Hispanic	Ref	Ref	Ref	Ref	Ref	Ref
Asian non-Hispanic	1.0 (0.74–1.3)	1.0 (0.75–1.4)	1.1 (0.73–1.5)	0.75 (0.51–1.1)	0.57 (0.33–0.97)	0.78 (0.41–1.5)
Black non-Hispanic	1.30 (0.89–1.90)	1.50 (1.10–2.00)	1.30 (0.99–1.70)	1.30 (1.00–1.60)	1.50 (1.20–1.80)	1.20 (0.83–1.80)
Hispanic	1.0 (0.67–1.6)	1.5 (1.0–2.3)	1.2 (0.81–1.7)	0.79 (0.56–1.1)	1.0 (0.78–1.4)	1.5 (0.94–2.3)
Other	0.81 (0.63–1.0)	1.3 (1.1–1.7)	0.87 (0.69–1.1)	1.2 (0.91–1.5)	0.99 (0.76–1.3)	1.0 (0.76–1.3)
Unknown	(0.95 (0.78–1.2)	1.0 (0.86–1.3)	1.0 (0.86–1.3)	1.0 (0.82–1.2)	0.84 (0.67–1.1)	1.0 (0.75–1.4)
**Insurance type**						
Commercial health plan	Ref	Ref	Ref	Ref	Ref	Ref
Medicare/other government plan	1.60 (1.30–2.00)	1.40 (1.20–1.70)	1.50 (1.20–1.80)	1.40 (1.10–1.70)	0.96 (0.78–1.20)	1.80 (1.30–2.40)
Medicaid	0.71 (0.35–1.4)	1.0 (0.59–1.7)	0.68 (0.40–1.2)	1.0 (0.66–1.6)	0.94 (0.61–1.5)	0.78 (0.39–1.6)
Others	0.84 (0.58–1.2)	1.0 (0.73–1.4)	0.67 (0.50–0.90)	0.87 (0.64–1.2)	0.96 (0.69–1.3)	1.2 (0.72–1.9)
Unknown	1.1 (0.93, 1.3)	1.2 (1.0–1.4)	1.3 (1.1–1.5)	1.2 (1.0–1.4)	1.1 (0.89–1.2)	1.5 (1.2–1.9)
**Practice type**						
Community	Ref	Ref	Ref	Ref	Ref	Ref
Academic	2.50 (2.10–2.90)	2.50 (2.10–2.90)	2.60 (2.20–3.10)	2.00 (1.60–2.40)	1.90 (1.50–2.40)	2.00 (1.40–2.60)

Abbreviations: CI, confidence interval; HR, hazard ratio; NA, not available; SES, socioeconomic status.

© 2026 Chehade CH et al. *JAMA Network Open*.

eFigure 1. Study Cohort

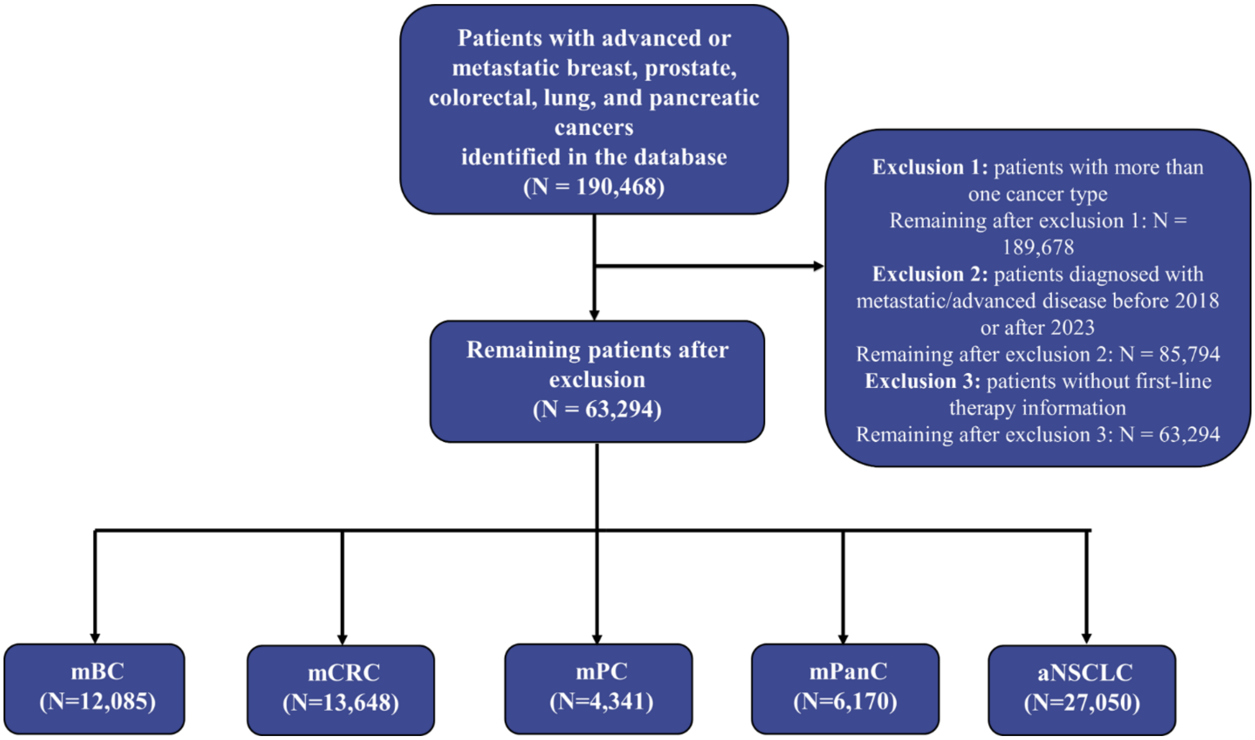

© 2026 Chehade CH et al. *JAMA Network Open*.

eFigure 2. Cumulative Incidence Functions of NGS Testing and Death by Cancer Type Following Metastatic or Advanced Diagnosis

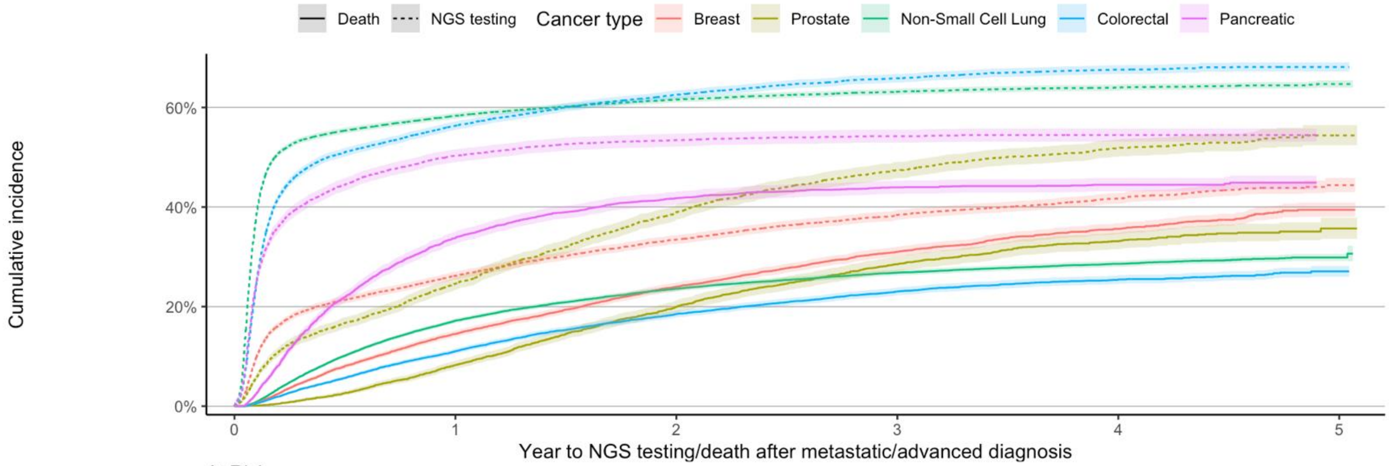

At Risk	0	1	2	3	4	5
Breast	12085	5884	3204	1586	607	15
Prostate	4341	2666	1359	615	227	7
Non-Small Cell Lung	27050	5564	2634	1264	465	11
Colorectal	13648	3850	1736	713	225	3
Pancreatic	6170	787	177	44	14	0

Events	0	1	2	3	4	5
Breast	0	4642	6122	6889	7216	7319
Prostate	0	1384	2340	2842	3033	3074
Non-Small Cell Lung	0	19921	21911	22660	22914	22982
Colorectal	0	8968	10433	11051	11264	11301
Pancreatic	0	4910	5405	5501	5516	5518

Shading represents the 95% CIs.

Abbreviations: NGS, next-generation sequencing.

© 2026 Chehade CH et al. *JAMA Network Open*.
