# Supplementary material for: Trends and Disparities in the Use of Next-Generation Sequencing in Patients With Cancer in the United States
Source: JAMA Netw Open. 2026 Apr 7;9(4):e265585. doi: 10.1001/jamanetworkopen.2026.5585 (PMC13058762; doi:10.1001/jamanetworkopen.2026.5585)
Supplement: Supplement 2. — Data Sharing Statement [file jamanetwopen-e265585-s002.pdf]

Data Sharing Statement

Chehade. Trends and Disparities in the Use of Next-Generation Sequencing in Patients with Cancer in the United States. *JAMA Netw Open*. Published April 07, 2026.
doi:10.1001/jamanetworkopen.2026.5585

Data
Data available: No

Additional Information
Explanation for why data not available: Data available: The data that support the findings of this study were originated by and are the property of Flatiron Health, Inc. Requests for data sharing by license or by permission for the specific purpose of replicating results in this manuscript can be submitted to PublicationsDataAccess@flatiron.com.
